# Exact model-free function inference using uniform marginal counts for null population

**DOI:** 10.1093/bioinformatics/btaf121

**Published:** 2025-03-20

**Authors:** Yiyi Li, Mingzhou Song

**Affiliations:** Department of Computer Science, New Mexico State University, Las Cruces, NM 88003, United States; Department of Computer Science, New Mexico State University, Las Cruces, NM 88003, United States; Molecular Biology and Interdisciplinary Life Sciences Graduate Program, New Mexico State University, Las Cruces, NM 88003, United States

## Abstract

**Motivation:**

Recognizing cause–effect relationships is a fundamental inquiry in science. However, current causal inference methods often focus on directionality but not statistical significance. A ramification is chance patterns of uneven marginal distributions achieving a perfect directionality score.

**Results:**

To overcome such issues, we design the uniform exact function test with continuity correction (UEFTC) to detect functional dependency between two discrete random variables. The null hypothesis is two variables being statistically independent. Unique from related tests whose null populations use observed marginals, we define the null population by an embedded uniform square. We also present a fast algorithm to accomplish the test. On datasets with ground truth, the UEFTC exhibits accurate directionality, low biases, and robust statistical behavior over alternatives. We found nonmonotonic response by gene *TCB2* to beta-estradiol dosage in engineered yeast strains. In the human duodenum with environmental enteric dysfunction, we discovered pathology-dependent anti-co-methylated CpG sites in the vicinity of genes *POU2AF1* and *LSP1*; such activity represents orchestrated methylation and demethylation along the same gene, unreported previously. The UEFTC has much improved effectiveness in exact model-free function inference for data-driven knowledge discovery.

**Availability and implementation:**

An open-source R package “UniExactFunTest” implementing the presented uniform exact function tests is available via CRAN at doi: 10.32614/CRAN.package.UniExactFunTest. Computer code to reproduce figures can be found in supplementary file “UEFTC-main.zip.”

## 1 Introduction

Causality is central to scientific inquiries, such as in biology and medicine. Knowing cause and effect allows one to characterize mechanisms. Mutations in *BRCA1* and *BRCA2* genes are causative factors for breast and ovarian cancers (Venkitaraman 2014). DNA methylation affects European lobster ageing (Fairfield *et al.* 2021), but not the other way round. Ageing and inflammation are highly correlated (Chung *et al.* 2009), yet how they are causally related remains unanswered (Jenny 2012, Pezone *et al.* 2023).

In contrast to experimental approaches that learn causality by perturbation, statistical causal inference often applies the causality-by-functionality principle (Simon and Rescher 1966) to observational studies. Conditional entropy (CE) is the expected Shannon entropy of discrete random variable Y given discrete random variable X (Shannon 1948). CE has been used for model-free function inference, without assuming a parametric form: it is minimized to zero if and only if Y is a function of X. Its critical weakness is optimality on both constant and nonconstant functions. Evidently, a constant function does not support a causal relationship. For this reason, CE is not routinely used in statistical practice, though widely covered in textbooks. Other approaches attempt to improve. The fraction of information (FOI) (Cavallo and Pittarelli 1987) evaluates Y’s entropy change given *X*. Goodman–Kruskal’s tau (GKT) (Goodman and Kruskal 1963) is the proportion of Y’s variability explained by X. Still, these effect-size quantities, more mathematical than statistical, are insufficient to answer how likely the quantities are observed if X and Y are independent. They tend to exaggerate a chance functional pattern with nonuniform marginals.

On the other hand, statistically equipped association testing such as Pearson’s chi-squared test (Pearson 1900), Fisher’s exact test (Fisher 1922), and G-test (Woolf 1957) is symmetrical and thus cannot infer function direction. Additionally but importantly, they use a null population whose marginal distributions follow the observed contingency table formed by X and Y. In modern applications where numerous tables have to be prioritized, *P*-values of tables from such a test are not comparable, because each *P*-value is relative to a specific null population, leading to increased false positives promoted by nonuniformity in table marginals.

Recent efforts start to improve model-free function inference. The functional chi-squared test (FunChisq or FC) (Zhang and Song 2013) uses a null population with the observed marginal for X and the uniform marginal for Y to establish a null distribution where statistical significance can be approximated. However, FC is biased to landscape tables (Y having more discrete levels than X) over portrait tables (Y having fewer discrete levels than X). Using FC’s test statistic, the exact function test (EFT) derives an exact null distribution with its null population defined by observed marginal counts (Zhong and Song 2018). Although EFT does not present strong table-shape biases, it tends to be equivocal about directionality. Adjustments on FC’s test statistic have been made to correct such biases (Kumar and Song 2022), but the *P*-value calculation remains inexact.

To overcome these challenges, we introduce the uniform exact function test with continuity correction (UEFTC) to infer function dependency on discrete data. In contrast to EFT, UEFTC uses a null population of uniform marginal counts; a square table is embedded in the original table. The *P*-value of an observed table is defined by the proportion of null tables with test statistic larger than or equal to the observed test statistic. Furthermore, for small-sized tables, the number of null tables is low, leading to a low resolution in *P*-value. To overcome this issue, we introduce continuity correction on the *P*-value. Since exact *P*-value calculation implies an exponential-time algorithm, we present a fast algorithm that extends an accelerated EFT algorithm (Nguyen *et al.* 2020), using branch-and-bound with dynamic programming for table enumeration.

We comprehensively evaluate UEFTC and other methods for function direction, table-shape bias, and function strength. Function direction accuracy is evaluated on the cause–effect pair dataset (Mooij *et al.* 2016), which contains both real and simulated examples with ground truth. We also simulated data of various table sizes, sample sizes, and marginal distributions under varying noise levels. They amount to hundreds of configurations and millions of tables to help us understand table-shape biases and function strength accuracy. UEFTC shows an overall advantage, while other methods demonstrate deficiencies in at least one aspect.

We applied UEFTC and other methods to identify functional relations in two biological studies. The first measured gene expression in engineered yeast strains responding to beta-estradiol dosage (Arita *et al.* 2021). The second quantified methylation and expression of genes in the duodenum of children with environmental enteric dysfunction (Haberman *et al.* 2021). UEFTC prioritized functional relations that are well supported statistically, while other methods favored functional patterns likely arising by chance or from outliers. Most surprisingly, we present anti-co-methylation among CpG sites in the vicinity of a gene; these patterns are associated with both gene expression and disease status. Although co-methylation is known (Guo *et al.* 2017) and detectable (Gatev *et al.* 2020), anti-co-methylation seems previously unreported in the literature. These results indicate the potential of UEFTC for function inference to support the discovery of causal relationships in scientific research.

## 2 Materials and methods

We address the hypothesis testing problem of function inference, which quantifies the extent random variable Y being a function of random variable X based on observed values of X and Y. Mathematically, Y is a function of X if and only if each value of X maps to a single value of Y. Here, we focus on X and Y being random discrete variables. We offer three methods that are model free—they do not assume a parametric functional form from X to Y. They are closely related statistical tests and vary in theoretical and efficiency considerations, all distinct from other existing tests in the design of null population.

### 2.1 Null hypothesis and test statistic

We define the null hypothesis $H_{0}$ that discrete random variables X and Y are statistically independent. To quantify the function dependency of Y on X, all three tests share the same functional chi-squared test statistic (Zhang and Song 2013). We represent joint (X,Y) observations by a contingency table O, where X and Y are the row and column variables, respectively. Let r and s be the numbers of rows and columns in the table, respectively. Let N be the total count. Let $n_{i\cdot }$ be the sum of row i. Let $n_{\cdot j}$ be the sum of column j. Let $n_{ij}$ be the entry of O at row i and column j. The FunChisq statistic is first defined by (Zhang and Song 2013) and later studied in (Nguyen 2018, Zhong and Song 2018, Nguyen *et al.* 2020):

(1) $$\chi_{f}^{2}(O)=\sum_{i=1}^{r}\sum_{j=1}^{s}\frac{{\left(n_{ij}-n_{i\cdot }/s\right)}^{2}}{n_{i\cdot }/s}-\sum_{j=1}^{s}\frac{{\left(n_{\cdot j}-N/s\right)}^{2}}{N/s}$$

It has been demonstrated that $\chi_{f}^{2}$ is maximized if and only if Y is a function of X given Y marginal counts (Zhang and Song 2013, Nguyen 2018). $\chi_{f}^{2}$ is minimized to zero if X and Y are empirically independent (Zhang and Song 2013). Using a null population with Y being uniform, the statistic asymptotically follows a chi-squared distribution (Zhang and Song 2013).

In practice, FC works well for distinguishing function from independence, but it often favors a function from a variable of fewer levels to another of more levels over the inverse. We call this a direction bias. We offer solutions to reduce such biases next.

### 2.2 A uniform multinomial function test

We argue that direction biases can arise from the choice of null population by a function inference method. Thereby, we study new null populations defined differently from classical ones based on observed marginals. We require that a null population must be equivalent when testing Y=f(X) on table O and X=f(Y) on the transposed table $O^{⊤}$. One such population is the *embedded uniform square*, where the population follows a joint uniform distribution on a k×k square subtable where k=min(r,s), with marginal probabilities $p_{i\cdot }$ of row i and $p_{\cdot j}$ of column j given by


(2) $$\begin{array}{c} p_{i\cdot }=\mathrm{Pr}(X=i)=\{\begin{array}{cc} 1/k & 1\leq i\leq k\\ 0 & k<i\leq r \end{array}\\ p_{\cdot j}=\mathrm{Pr}(Y=j)=\{\begin{array}{cc} 1/k & 1\leq j\leq k\\ 0 & k<j\leq s \end{array} \end{array}$$

Let A be the set of all r×s tables with the same sample size N. Under the null hypothesis, counts in tables in A follow a multinomial distribution. So, the null probability of table A∈A is


(3) $$\mathrm{Pr}(A)=\frac{N!}{\prod_{i=1}^{r}\prod_{j=1}^{s}n_{ij}!}\prod_{i=1}^{r}\prod_{j=1}^{s}{\left(p_{i\cdot }p_{\cdot j}\right)}^{n_{ij}}$$

Thus, the *P*-value of observed table O is defined by


(4) $$p–\text{value}(O)=\sum_{\chi_{f}^{2}(A)\geq \chi_{f}^{2}(O)}\mathrm{Pr}(A),\;\text{where}\;A\in A$$

which constitutes the uniform multinomial function test (UMFT).

Although the null test statistics of $\chi_{f}^{2}(A)$ and $\chi_{f}^{2}(A^{⊤})$ are not directly comparable, they follow mathematically equivalent distributions and thus overcoming direction biases. UMFT is exact in its *P*-values. However, its time complexity is $O({\left(rs\right)}^{N})$, exponential in sample size due to table enumeration. So, UMFT provides a gold standard for small tables of moderate counts.

### 2.3 A uniform exact function test

To approximate UMFT at a lower computational cost, we reduce the null population to include only tables with uniform row and column sums. Using this null population, we introduce the uniform exact function test (UEFT). Let B be the null population of all r×s tables of sample size N with uniform row and column sums. As N cannot always be divided by r and s with a zero remainder, we define approximately equal counts as uniform row sums by $R^{0}=(R_{1},\ldots ,R_{r})$ where


(5) $$R_{i}=\{\begin{array}{cc} \left\lceil N/k\right\rceil & 1\leq i\leq (N\;\text{mod}\;k)\\ \left\lfloor N/k\right\rfloor & (N\;\text{mod}\;k)<i\leq k\\ 0 & k<i\leq r \end{array}$$

and uniform column sums by $C^{0}=(C_{1},\ldots ,C_{s})$ where


(6) $$C_{j}=\{\begin{array}{cc} \left\lceil N/k\right\rceil & 1\leq j\leq (N\;\text{mod}\;k)\\ \left\lfloor N/k\right\rfloor & (N\;\text{mod}\;k)<j\leq k\\ 0 & k<j\leq s \end{array}$$

Under the null hypothesis of row (X) and column (Y) being statistically independent, the probability of B is given by the hypergeometric distribution:


(7) $$\mathrm{Pr}(B)=\frac{\prod_{i=1}^{r}R_{i}\prod_{j=1}^{s}C_{j}!}{N!\prod_{i=1}^{r}\prod_{j=1}^{s}n_{ij}!}$$

To detect functional patterns, we use the upper tail of null distribution to calculate the *P*-value by


(8) $$p–\text{value}\;(O)=\sum_{\chi_{f}^{2}(B)\geq \chi_{f}^{2}(O)}\mathrm{Pr}(B),\;\text{where}\;B\in B$$

It is evident that B is a subset of A, the null population of UMFT, but it is not obvious that B can reach the same maximum statistic on A, as established by Theorem 1.

Theorem 1. *Let* A  *be the set of all* r×s  *tables of sample size* N*. Let* B  *be a subset of* A  *so that* B  *contains those tables in* A  *with uniform row and column sums. Both sets achieve the same maximum test statistic:*
 

(9) $$\underset{B\in B}{\max}\;\chi_{f}^{2}(B)=\underset{A\in A}{\max}\;\chi_{f}^{2}(A)$$

The proof and supporting lemmas are given in Supplementary Materials Section S1.

### 2.4 A uniform exact function test with continuity correction

UEFT is much faster than UMFT but comes with reduced function inference accuracy. Uniform null marginal sums limit null test statistic variations, making exact *P*-values discrete for small-sized tables: many observed tables with different test statistics can share the same *P*-value. To address this limitation, we introduce continuity correction to the exact *P*-value of UEFT. We call this changed test UEFTC.

**Figure 1.**
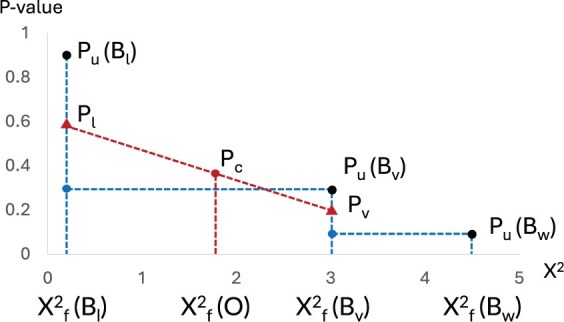
*P*-value continuity correction via interpolation. Neighbors of observed table O in test statistic (horizontal axis) are tables $B_{v}$, $B_{l}$, and $B_{w}$ in null population B, with exact *P*-values $P_{U}(B_{l})$, $P_{U}(B_{v})$, and $P_{U}(B_{w})$ (vertical axis). Smoothed *P*-values $P_{l}$ and $P_{v}$, of $B_{l}$ and $B_{v}$, respectively, are used to interpolate a *P*-values $P_{c}$ for table *O*.

As the test statistic of an observed table O is not always achievable in null population B, our solution is to smooth the exact *P*-values of null test statistics (achievable in B) surrounding the observed one. Specifically, we find three neighbor tables $B_{l}$, $B_{v}$, and $B_{w}$ from B such that


(10) $$\chi_{f}^{2}(B_{l})\leq \chi_{f}^{2}(O)\leq \chi_{f}^{2}(B_{v})<\chi_{f}^{2}(B_{w})$$

and no other test statistics from tables in B are in between but distinct from the four statistics in the equation. Let $P_{U}(B_{l})$, $P_{U}(B_{v})$, and $P_{U}(B_{w})$ be exact UEFT *P*-values [Equation (8)] of the three tables. Figure 1 illustrates *P*-value continuity correction via interpolation.

Given O, we first define its lower neighbor statistic $\chi_{f}^{2}(B_{l})$. If $\chi_{f}^{2}(O)\geq \underset{B\in B}{\min}\;\chi_{f}^{2}(B)$, we can always find some table $B_{l}\in B$ such that

(11) $$\chi_{f}^{2}(B_{l})=\max\left\{\chi_{f}^{2}(B)|\chi_{f}^{2}(B)\leq \chi_{f}^{2}(O),B\in B\right\}$$

In the case of $\chi_{f}^{2}(O)<\underset{B\in B}{\min}\;\chi_{f}^{2}(B)$, we define $\chi_{f}^{2}(B_{l})=0$ and $P_{U}(B_{l})=1$. Second, we define O’s upper neighbor statistic $\chi_{f}^{2}(B_{v})$. By Theorem 1, we can always find some table $B_{v}\in B$ such that

(12) $$\chi_{f}^{2}(B_{v})=\min\left\{\chi_{f}^{2}(B)|\chi_{f}^{2}(B)\geq \chi_{f}^{2}(O),B\in B\right\}$$

which implies $O^{\prime }s$ UEFT *P*-value $P_{U}(O)=P_{U}(B_{v})$. Third, we define O’s super neighbor statistic $\chi_{f}^{2}(B_{w})$ that must be greater than $\chi_{f}^{2}(B_{v})$. In the case that $\chi_{f}^{2}(B_{v})$ is the maximum test statistic in B, we define $P_{U}(B_{w})=0$. Otherwise, we can always find some table $B_{w}\in B$ such that

(13) $$\chi_{f}^{2}(B_{w})=\min\left\{\chi_{f}^{2}(B)|\chi_{f}^{2}(B)>\chi_{f}^{2}(B_{v}),B\in B\right\}$$

By averaging the exact *P*-values at the boundaries, we correct *P*-values of $B_{l}$ and $B_{v}$ by

(14) $$P_{l}=\frac{P_{U}(B_{l})+P_{U}(O)}{2}\;\text{and}\;P_{v}=\frac{P_{U}(O)+P_{U}(B_{w})}{2}$$

which form a pair of neighboring points

(15) $$\left(\chi_{f}^{2}(B_{l}),P_{l}\right)\;\text{and}\;\left(\chi_{f}^{2}(B_{v}),P_{v}\right)$$

In the boundary case of $\chi_{f}^{2}(O)=0$, both the exact and corrected *P*-values are 1. In the case of $\chi_{f}^{2}(O)$ equaling a null test statistic, we must have $\chi_{f}^{2}(B_{l})=\chi_{f}^{2}(B_{v})$ and assign $P_{v}$ to $O^{\prime }s$  *P*-value. Otherwise, we calculate the corrected *P*-value by linear interpolation at $\chi_{f}^{2}(O)$ using the two neighboring points. Taking altogether, we obtain $P_{c}$, the continuity corrected *P*-value of O, by

(16) $$P_{c}=\{\begin{array}{cc} 1 & \chi_{f}^{2}(O)=0\\ P_{v} & \chi_{f}^{2}(B_{l})=\chi_{f}^{2}(O)=\chi_{f}^{2}(B_{v})\neq 0\\ P_{l}+\frac{\chi_{f}^{2}(O)-\chi_{f}^{2}(B_{l})}{\chi_{f}^{2}(B_{v})-\chi_{f}^{2}(B_{l})}\cdot (P_{v}-P_{l}) & \text{otherwise} \end{array}$$

which defines the *P*-value of the UEFTC test.

#### 2.4.1 Improved null distribution

**Figure 2.**
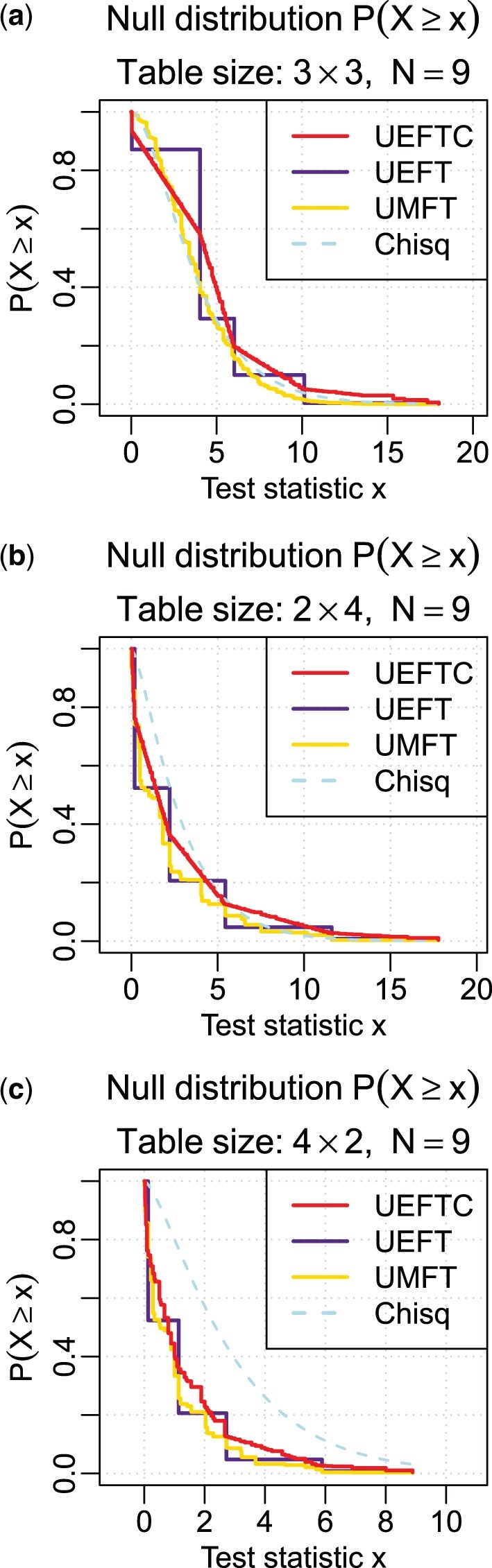
Null test statistic distributions of UEFTC, UEFT, and UMFT. The chi-squared approximation (Chisq) is also provided for a contrast. Horizontal axes are test statistic. Null distributions shown are P(X≥x) (vertical axis), instead of the cumulative distribution, to directly correspond to the *P*-values. The null population consists of all tables with given sample size (*N *=* *9) and shapes. (a) 3×3 square tables. (b) 2×4 landscape tables. (c) 4×2 portrait tables.

To understand the effect of continuity correction on the null distribution, we obtained *P*-values of UMFT, UEFT, UEFTC, and the chi-squared distribution (used by FC) for all possible 3×3, 2×4, and 4×2 tables with sample size 9 (Fig. 2). UEFTC visibly improved *P*-value continuity with a null distribution closer to UMFT than UEFT. The chi-squared distribution is close to UMFT for square tables; however, it deviates from UMFT for landscape tables more than the other tests; most dramatically, it over-estimates *P*-values for portrait tables.

#### 2.4.2 Improved function directionality

**Figure 3.**
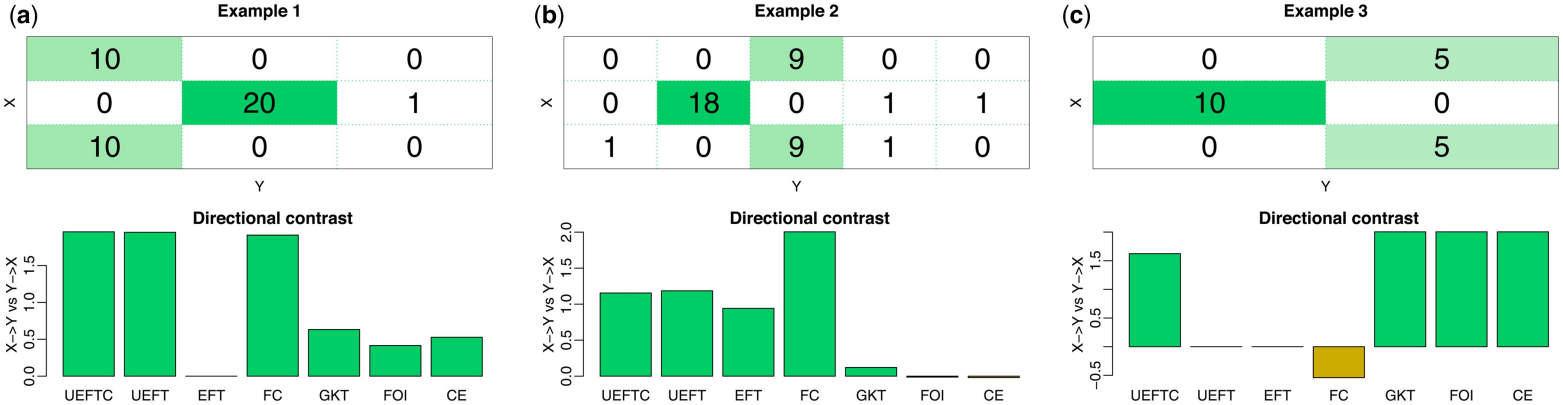
Direction contrast of function inference methods. Two noisy and one perfect many-to-one function patterns Y=f(X) cover (a) square, (b) landscape, and (c) portrait tables. Bar plots (bottom) show log ratios of test results between X→Y and Y→X, defined by −log(ratio+0.01)+log(1.01), so that it is 0 if and only if test results are equal for both directions. A positive/negative bar indicates a correct/wrong direction, respectively.

To show the impact of continuity correction on function direction inference, we illustrate table-shape biases of seven methods (UEFTC, UEFT, EFT, FC, GKT, FOI, and CE) on nonmonotonic functions whose direction is unambiguous. As the examples (Fig. 3) are at low noise levels, a method failing on any suggests a serious deficiency. The first four methods are evaluated by *P*-values and by test statistics for GKT, FOI, and CE. Direction contrast, or the log ratio of test results between X→Y and Y→X, is used to evaluate performance: a positive value indicates a correct direction decision. UEFT obtained correct directions on square and landscape tables but is ambiguous on the portrait table (Fig. 3c). UEFTC, fixing UEFT’s deficiency, shows high direction contrasts for all function patterns. Although GKT also identified correct directions for all examples, its direction contrast is much lower in the landscape table, suggesting a potential bias. In contrast, EFT only rated the landscape function high but is ambiguous about the other two (Fig. 3a and c). FC disqualified the portrait function. FOI and CE are ambiguous for the landscape function (Fig. 3b), carrying strong biases favoring portrait tables.

### 2.5 Computing the exact P value quickly

To speed up UEFT and UEFTC, we adapt a branch-and-bound method introduced earlier for EFT (Nguyen *et al.* 2020). The main change is to modify EFT’s null population based on observed marginal counts to the embedded uniform square—the null population B defined for UEFT and UEFTC. Neighboring statistics of an observed test statistic are also returned to support continuity correction. The fast computation integrates dynamic programming into reducing branches in *P*-value calculation (Supplementary Fig. S1). Method design (Supplementary Algorithms S1–S4) and runtime (Supplementary Fig. S2) are presented with full details in Supplementary Materials Section S2.

## 3 Results

### 3.1 Performance evaluation

To quantify how well a method performs function inference, we identified or simulated datasets with ground truth. Specifically, we compared UEFT and UEFTC with other model-free methods, including FC, EFT, CE, FOI, and GKT. Fisher’s exact test and G-test are also included to study function strength accuracy; however, both are symmetric tests and thus are excluded from the function directionality study. UMFT is omitted for its prohibitive runtime.

#### 3.1.1 Overall performance summary

**Figure 4.**
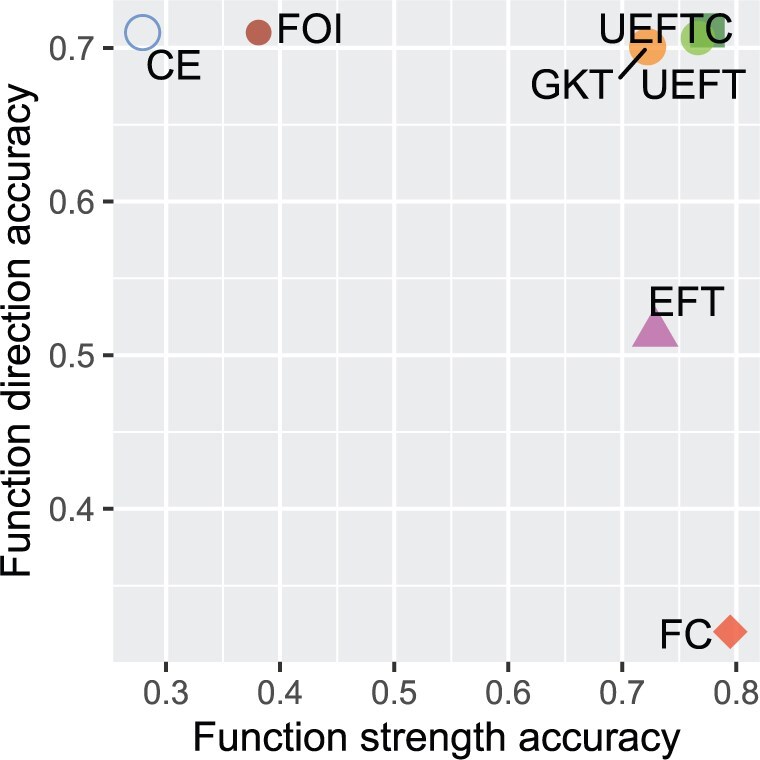
Overall performance of seven model-free function inference methods. Function direction accuracy (vertical axis) is the probability of correct identification using cause–effect pair data. Function strength accuracy (horizontal axis) is the AUPR obtained on simulated functional versus independent tables.

We evaluated the methods by both strength and direction of function. For strength on functional Y=f(X) versus independent X⊥Y patterns, the criterion is area under the precision-recall curve (AUPR) on simulated tables with various sample sizes, shapes, marginal distributions, and noise levels. For function direction on X→Y versus Y→X, we assessed the percentage of correct direction on the cause–effect pair dataset (Mooij *et al.* 2016) including both real-world and simulated examples. Fisher’s exact test and G-test are excluded as they do not assess function direction. The overall performance is summarized in Fig. 4. Both UEFTC and UEFT stand out without major deficiencies in either criterion. Specifically, UEFTC is the best in function direction accuracy and the second best in functional strength accuracy. Following UEFTC and UEFT, GKT performed well but is subject to outliers (see top but spurious patterns picked by GKT in Fig. 10). While FC and EFT performed well in function strength, they have deficiencies in function direction; in contrast, CE and FOI behaved well in function direction but poorly in function strength. Therefore, our evaluation provides evidence that UEFTC and UEFT have advanced model-free function inference.

#### 3.1.2 Function strength accuracy

**Figure 5.**
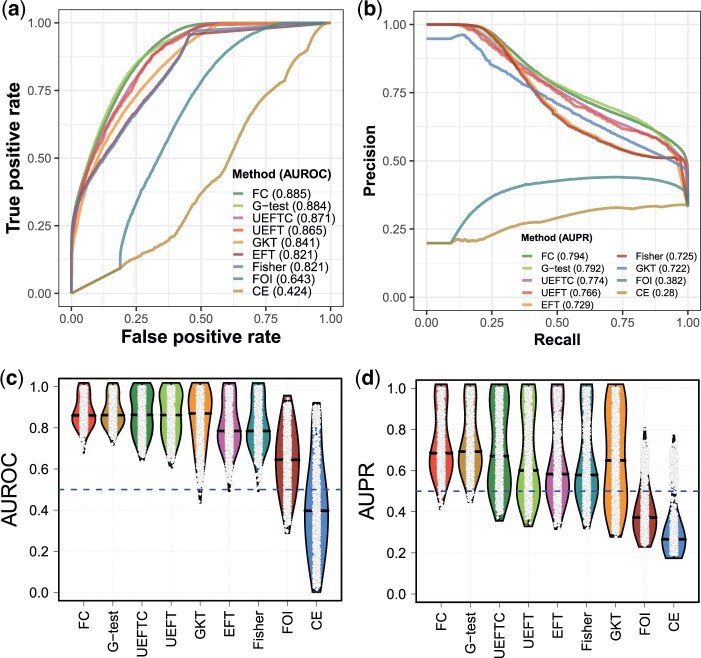
Performance of nine methods on distinguishing functional from independent patterns. (a, b): ROC and PR curves of each method for 420 000 simulated tables. (c, d): Violin plots of AUROC and AUPR distributions for each method, ordered by the mean of AURPC/AUPR. A white point represents a table setup. The black bar within each violin plot is the median of AUROC or AURP values.

To assess methods on inferring function strength, we simulated noisy functional (Y=f(X)) and independent (X⊥Y) patterns and generated receiver-operating characteristic (ROC) and precision-recall curves. Using the simulate_tables() function (Sharma *et al.* 2017) in R package “FunChisq,” we generated 280 000 independent tables with five table sizes, ten sample sizes, and uniform or nonuniform marginal distributions (Supplementary Table S1). The nonuniform marginal distribution is defined by $\mathrm{Pr}(i)=i^{8}/\sum_{m=1}^{M}m^{8}$, where i is row/column index, and M is the number of rows/columns. Next, we simulated 140 000 functional tables at seven noise levels (Zhang *et al.* 2015), various table sizes, and sample sizes (Supplementary Table S1); here, Y’s marginal cannot be configured because Y is a function of X and its marginal is determined by X’s marginal. We applied each method to rank tables in each setup so as to produce ROC and PR curves and collect AUROC and AUPR values. FunChisq and G-test achieved the best functional strength accuracy with the best ROC and PR curves (Fig. 5a and b) and the highest mean AUPR and AUROC including all setups (Fig. 5c and d). Following G-test closely, UEFTC obtained the third highest AUROC and AURP, better than UEFT, indicating that our continuity correction is effective. The precision of GKT at a low recall is not ideal. FOI and CE performed the worst—close to random guessing.

The marginal distribution has a major impact on function inference, as shown by Supplementary Fig. S3. We simulated tables grouped by marginal distributions using four configurations in Supplementary Tables S2–S5. At uniform row and column marginals, all methods are comparable but at relatively low functional strength accuracy in contrast to other configurations. When one marginal is nonuniform, only UEFTC, UEFT, G-test, and FC do not demonstrate evident deficiencies, while GKT, EFT, Fisher, FOI, and CE under-performed substantially in some configurations.

**Figure 6.**
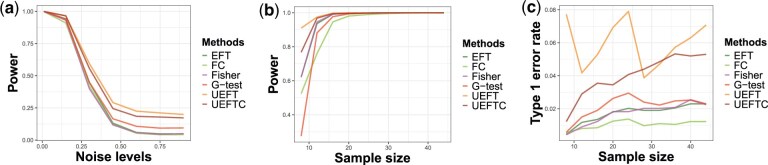
Performance of six methods at various sample sizes and noise levels. (a) Statistical power over noise levels. (b) Statistical power over sample sizes. (c) Type 1 error rate over sample sizes.

We also examined the statistical power and type 1 error of the methods, while fixing the declared significance level at 0.05. GKT, FOI, and CE are excluded as they do not provide *P*-values. We studied the statistical power under noise levels 0.01, 0.15, 0.3, 0.45, 0.6, 0.75, and 0.9, and sample sizes 8, 12, 16, 20, 24, 28, 32, 36, 40, 44, in two configurations as described in Supplementary Tables S6 and S7. Figure 6a and b shows that UEFT, followed by UEFTC, achieved the highest power (over both noise levels and sample sizes) among all methods. In Fig. 6c, the type 1 error rate of UEFT is the highest, while UEFTC is closest to 0.05, the declared significance level.

#### 3.1.3 Function direction accuracy

To assess methods on function direction inference, we used 70 nonmonotonic functional patterns from the cause–effect pair dataset (Mooij *et al.* 2016). Data variables were scaled by standard deviation. We used spherical Gaussian mixture models (“VII”) of 2–7 components via R package “mclust” v6.0.0 (Scrucca *et al.* 2016) to find clusters, based on which we discretized the data to create contingency tables by R package “GridOnClusters” v0.1.7 (Wang *et al.* 2020). To save CPU cycles, we randomly chose 50 points from each table. We repeated the sampling 50 times to obtain performance ranges. The direction of a table is determined by the stronger statistic between X→Y (row to column) and Y→X (column to row).

**Figure 7.**
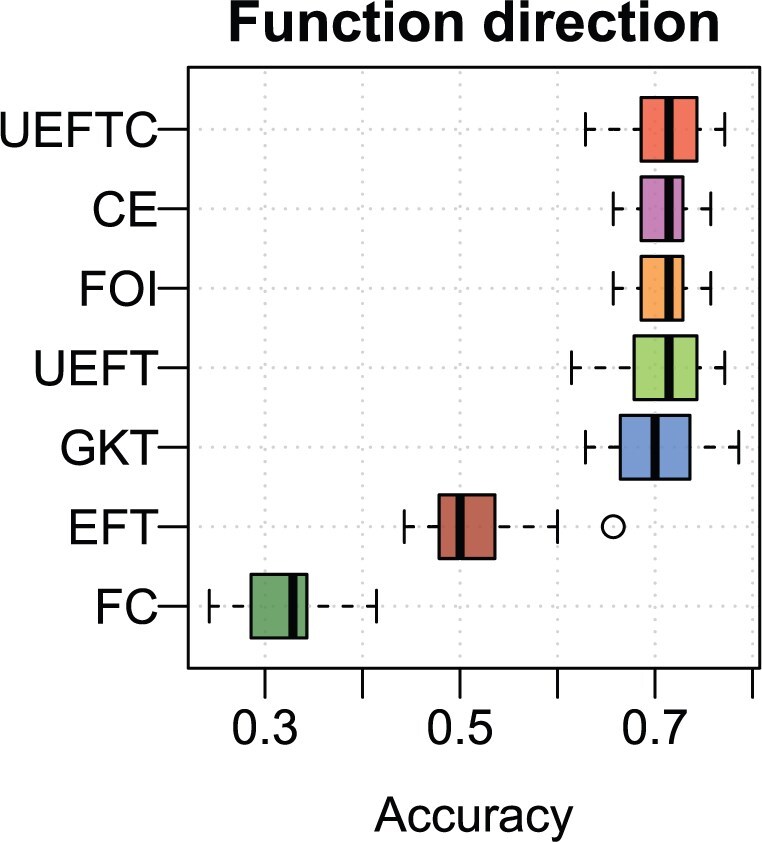
Performance on detecting function direction. Box plots show fractions of correct predictions (horizontal axis) using randomly sampled cause–effect pair data by the seven methods (vertical axis). The thick black bar inside each box is median fraction.

**Figure 8.**
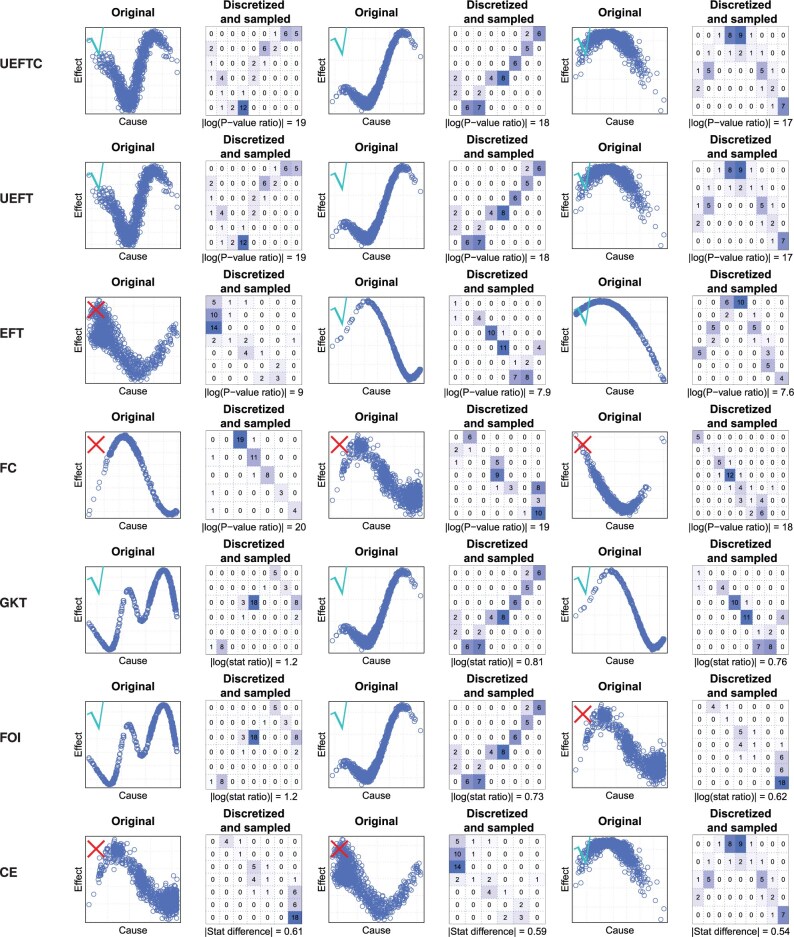
Top three cause–effect patterns detected by each method. Patterns are ordered by absolute values of log-*P*-value ratios (UEFTC, UEFT, EFT, FC), log statistic ratios (GKT, FOI), or difference in statistic (CE). A check/X marks a correct/wrong causal direction.

Fractions of correct directions are shown in Fig. 7. UEFTC outperformed other methods. Condition entropy, FOI, UEFT, and GKT followed. The median of EFT’s correct direction fraction is close to 0.5, suggesting loss of directionality. FC underperformed. In Fig. 8, we show the top three patterns returned by each method by difference in *P*-values or statistics between X→Y and Y→X directions. UEFCT, UEFT, and GKT are correct on their top three patterns. EFT and FOI are correct on two. Condition entropy has only one correct direction. FC is incorrect on all three. More importantly, the top three patterns differ substantially among the methods, suggesting fundamental differences in principle.

#### 3.1.4 Biases over function direction

**Figure 9.**
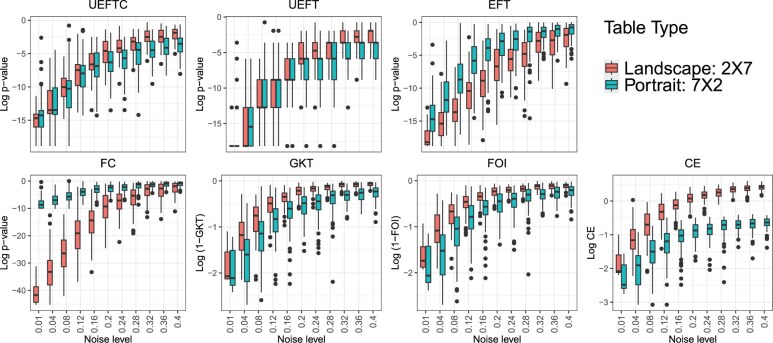
Biases over table shapes by seven methods. Landscape (2×7) versus portrait (7×2) functional tables subject to increasing noise levels (horizontal axes) are evaluated by each method. Vertical axes are log-scores of each method; lower values indicate stronger functions.

Table-shape biases refer to that a method tends to favor either landscape (fewer rows than columns) over portrait (more rows than columns) tables or the other way round—a long-recognized issue, see Kumar and Song (2022) for example. To study how table-shape biases influence a method, we used R package “FunChisq” v2.5.3 to generate 100 functional landscape and portrait tables randomly with sample size 30 at noise levels from 0.01 to 0.4. We study score variations of each method to assess table-shape biases. If biases are absent, the score distributions are expected to be similar between both table shapes. Figure 9 contrasts score distributions between landscape and portrait functional patterns for each method.

At low noise levels from 0.01 to 0.16, tables represent strong functional patterns. UEFT’s *P*-values exhibit visible biases at noise levels ≥ 0.08. UEFTC, correcting *P*-value discontinuity, maintains minimal biases at all low noise levels. Other methods carry modest to high biases: FC and EFT prefer landscape tables, while FOI, CE, and GKT favor portrait tables.

At high noise levels from 0.2 to 0.4, functional patterns turn independent. Biases of FOI, FC, GKT, and EFT become lower, though CE is still strongly biased against landscape tables. UEFTC also carries slight biases against landscape tables. As nearly independent patterns will yield *P*-values close to 1, accurate direction on such statistically insignificant tables may no longer be meaningful. So low biases of UEFTC on stronger functions (low noise) make it the best choice.

### 3.2 Engineered yeast gene expression in response to beta-estradiol dosage

Dynamic gene expression can present functional patterns in response to stimuli. Here, we demonstrate how our approaches can prioritize genes showing interesting dose response in genetically engineered yeast strains, while other approaches may return uninteresting patterns. Via genetic engineering, artificial transcription factors called ZEVs become constitutive in modified yeast strains (Arita *et al.* 2021). By binding to synthetic promoters inserted upstream of thousands of open-reading frames (ORFs) in the yeast genome, ZEVs can induce ORF expression. Meanwhile, beta-estradiol dosage can control ZEV activity to manipulate gene expression.

**Figure 10.**
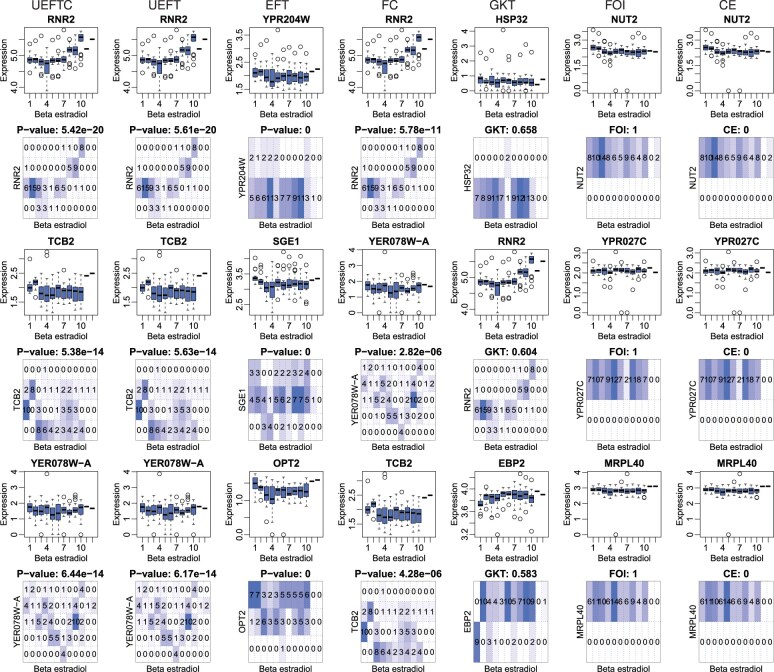
Top engineered yeast gene expression patterns in response to beta-estradiol dosage. Each column is a method and its top three genes. The horizontal axis represents 12 dosages of beta-estradiol, arranged from left to right as follows: two 0 nM dosages, 0.03125–0.125 nM, 0.5 nM, 1 nM, 4 nM, 16 nM, 64 nM, 100 nM, 1000 nM, and two additional HO strains treated with 1000 nM. Vertical axes are the original (odd row) or discretized (even row) gene expression levels. Patterns are ordered by scores from low to high (UEFTC, UEFT, EFT, FC, CE) or high to low (GKT, FOI).

We obtained RNA-seq data for 5655 genes from 19 yeast strains in response to multiple beta-estradiol dosages. The strains were selected according to 19 alleles, including *ANY1, ARO1, ASC1, ATG4, BAT1, CAJ1, CLN2, HO, MET2, MET6, SHM2, SNF12, SRP40, SRS2, SNT1, THR1, TIP41, TRP4*, and *VMA3*. There are a total of 192 samples with 10–12 samples per strain. Data were normalized to the log of transcripts per million (TPM), and further discretized to 2 to 7 levels by R package “Ckmeans.1d.dp” (Wang and Song 2011, Song and Zhong 2020). To reduce CPU cycles, 80 samples were randomly selected from discretized values for each gene. Top three patterns by each method are shown in Fig. 10.

The top three returned by UEFTC are *RNR2*, *TCB2*, and *YER078W-A*. *RNR2* showed a low-to-high dynamic expression pattern over dosage, expected by the experimental design. However, both *TCB2* and *YER078W-A* showed nonmonotonic responses. *TCB2* reached maximum expression at low dosage (0 nM), sharply dropped to minimum level, and then stayed at low and medium expression levels at high dosages; YER078W-A started with high expression, decreased to the minimum at dosage 16 nM, and eventually maximally expressed at the highest dosage. Thus, UEFTC recovered dynamic patterns invisible with the original data where outliers often dwarfed interesting expression dynamic.

UEFTC, UEFT, and FC obtained similar dynamic patterns that can be either monotonic or nonmonotonic. EFT’s top gene pattern seems weak from both the original and the discretized box plots. Figure 10 also reveals fundamental limitations of effect-sized based methods GKT, FOI, and CE. As demonstrated by the discretized patterns, they all promoted seemingly perfect functional patterns, most likely due to outliers causing uneven marginal (gene expression) distributions. Their top ranked patterns show either completely or nearly flat dose-response dynamics, making it highly questionable to use these methods on data with wide dynamic ranges.

Complementary to the original study (Arita *et al.* 2021) reporting monotonic dose response for all genes, our study, being model free, has thus picked up genes showing interesting nonmonotonic dose response.

### 3.3 Gene methylation-expression patterns dependent on pathology

**Figure 11.**
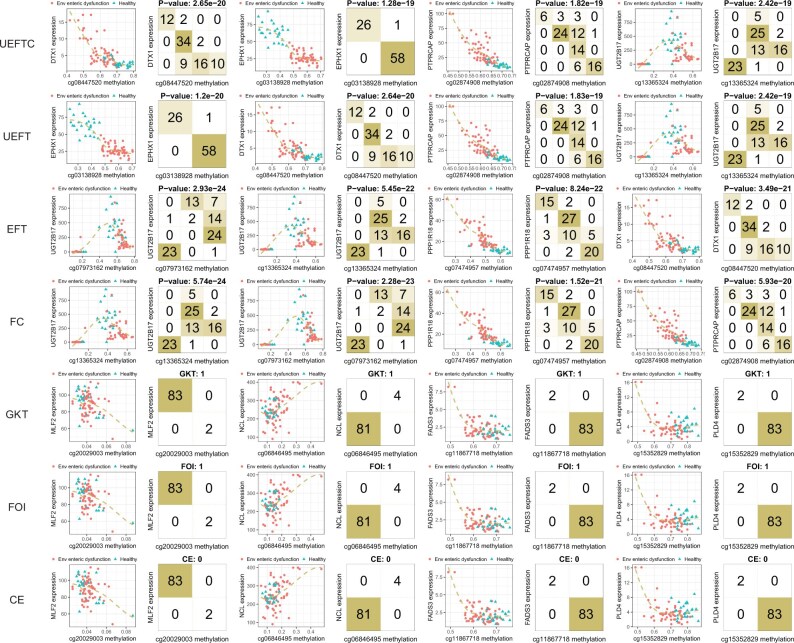
Top DNA methylation–gene expression patterns in human duodena with EED, as detected by seven methods. Environmental enteric dysfunction subjects are marked in dot and control subjects are in triangle. Each plot shows gene expression versus DNA methylation levels of a nearby CpG site. Vertical axes are original or discretized gene expression levels. Horizontal axes are original or discretized CpG-site methylation levels. Scores of each method are shown on top of each discretized pattern. Patterns are ordered from left to right by scores of each method from low to high (UEFTC, UEFT, EFT, FC, and CE) or high to low (GKT and FOI).

To further demonstrate how UEFTC may discover novel patterns, we applied UEFTC and other methods to study how gene expression is a function of DNA methylation in the context of environmental enteric dysfunction (EED). We obtained DNA methylation beta values of CpG sites and RNA-seq TPMs of genes in the duodenum from 52 children diagnosed with EED and 42 control subjects (Haberman *et al.* 2021). Data were scaled by standard deviation and then discretized by R package “mclust” v6.0.0 using spherical Gaussian mixtures (model “VII”) (Scrucca *et al.* 2016) and “GridOnClusters” v0.1.7 (Wang *et al.* 2020). We examined all possible 726 148 relationships from a CpG site’s methylation level to a nearby gene expression. Top four pairs by UEFTC, UEFT, FC, EFT, GKT, FOI and CE are shown in Fig. 11. Top four pairs from UEFTC and UEFT involve *DTX1*, *EPHX1*, *PTPRCAP*, and *UGT2B17*. The first three are positively associated with methylation levels of CpG sites, while *UGT2B17* expression is nonmonotonic to methylation levels of a CpG site. Subjects are clustered by disease status, despite such information not being used in ranking the pairs, suggesting strong disease-dependent variations in methylation-expression dynamic. *DTX1* (dinophysistoxin-1) is highly expressed in EED. A related marine lipophilic phycotoxin accumulates in the shellfish and may cause gastrointestinal illness (Louzao *et al.* 2021). *EPHX1* (microsomal epoxide hydrolase) functionally depends on cg03138928 *EPHX1_E152_F*, associated with inflammatory bowel disease (Lin *et al.* 2011). *PTPRCAP* is a methylation-driven key regulator related to gastric cancer (Yoo *et al.* 2020). *UGT2B17* forms a nonmonotonic pattern with cg19481811. DNA methyltransferase inhibitor *RG108* treatment decreases *UGT2B17* expression (Shafiee-Kermani *et al.* 2021) in prostate cancer, indicating positively related expression and methylation. Thus, all four genes are involved in either gastrointestinal function or methylation, supporting our findings. EFT and FC returned top four pairs arranged in different order and share three with UEFTC and UEFT. Top four pairs by FOI, CE, and GKT are identical but uninteresting, presenting a mixed subject cloud with outliers and no association with disease status.

**Figure 12.**
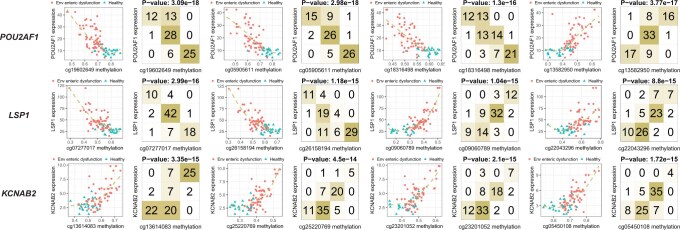
DNA methylation of multiple CpG sites in the vicinity of a gene is associated with gene expression and environmental enteric dysfunction. Horizontal axes are methylation levels of specific CpG sites. Vertical axes are expression of gene in the vicinity of a CpG site. CpG sites are ordered by their locations along each gene from 5′ to 3′ end.

**Figure 13.**
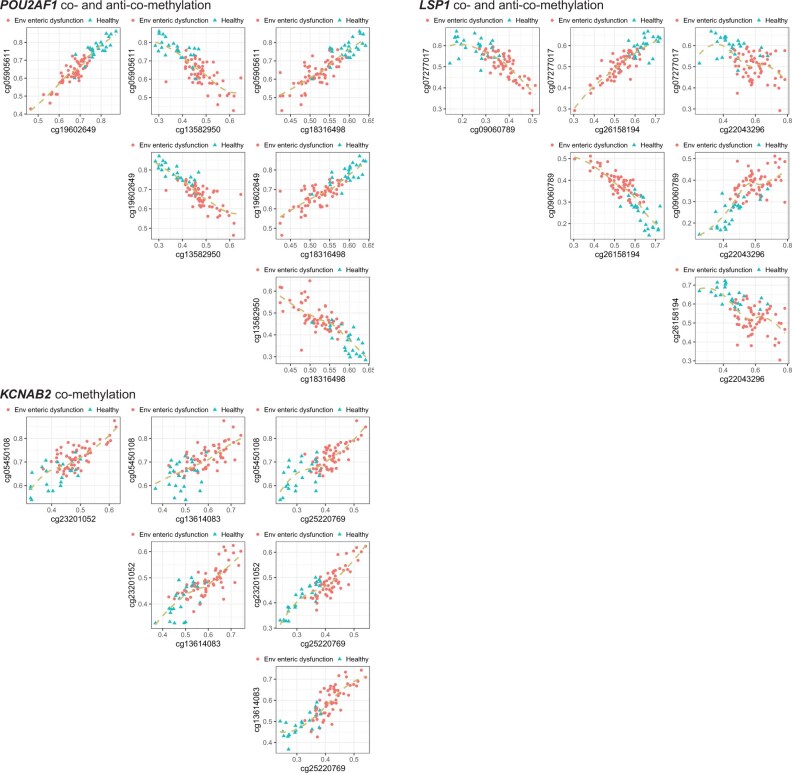
Anti-co-methylation and co-methylation among CpG sites in the vicinity of a gene and association with disease. Axes are DNA methylation levels of CpG-site pairs in the vicinity of a gene. Dots denote subjects with environmental enteric dysfunction and triangles are control subjects. CpG-site pairs are shown for three genes *POU2AF1*, *LSP1*, and *KCNAB2*. For each gene, CpG sites are found either upstream or in gene body.

Strikingly, we found orchestrated anti-co-methylation or co-methylation between CpG sites in the vicinity of a gene, associated with both gene expression and disease status, from UEFTC’s top 100 pairs. We showcase *POU2AF1*, *LSP1*, and *KCNAB2* (Figs 12 and 13), all highly expressed in duodena of EED subjects relative to the control. This is consistent with their low expression in healthy human duodena, but notably high expression in healthy lymph node, spleen, and appendix (Fagerberg *et al.* 2014).

*POU2AF1* (POU class 2 homeobox associating factor 1) hosts four CpG sites that are anti-co- or co-methylated. Sites cg19602649, cg05905611, and cg18316498, upstream of all annotated transcripts of *POU2AF1* (Kent *et al.* 2002), are all hypomethylated in EED, while being negatively associated with gene expression (Fig. 12). cg13582950, inside the last exon of a transcript, is hypermethylated in EED but negatively associated with gene expression (Fig. 12). Thus, the first three CpG sites form anti-co-methylation patterns with the last site (Fig. 13).

*LSP1*, lymphocyte specific protein 1, includes four CpG sites that are either anti-co- or co-methylated. cg07277017 is upstream of all annotated transcripts. cg26158194 is inside the first exon of two transcripts. cg09060789 is intronic to some transcripts but upstream of other transcripts. cg22043296 is in the first intron of some transcripts, the first exon of some transcripts, and also upstream of other transcripts.

*KCNAB2*, potassium voltage-gated channel subfamily A regulatory beta subunit 2, contains four CpG sites that are all hypermethylated in the EED subjects, while being positively associated with gene expression. cg13614083 is located upstream of all annotated transcripts of the gene. cg25220769 is more complex: it is within the intron of some transcripts and upstream of the start site of another transcript. cg23201052 is located in the intron of some transcripts. cg05450108 is in an intron of some transcripts and an exon of other transcripts. This situation is somehow unconventional for the lack of a hypomethylated promoter region to account for upregulation of gene expression in the EED subjects.

## 4 Discussion

The presented null distribution defined by embedded uniform squares allows us to use an equivalent distribution to evaluate the statistical significance of both portrait and landscape tables. This has greatly reduced table-shape biases in strong functional patterns even at low noise levels. We have shown that EFT and FC favor landscape tables, while GKT, FOI, and CE prefer portrait tables. We have proven that this null distribution does cover the maximum statistics possible. UEFTC further overcomes *P*-value discontinuity by smoothing exact *P*-values to fix exact but inaccurate *P*-values by UEFT due to a small null population size.

Our study on function direction inference shows that UEFTC outperformed all other methods. Our function strength study confirms that UEFTC does not lose power in function strength inference when compared with symmetrical methods including Fisher’s exact test and G-test, and other asymmetric methods. These make UEFTC the only viable option with a high functional direction accuracy. We designed fast algorithms to improve time efficiency of UEFT and UEFTC, achieving comparable runtime with other exact tests. However, runtime is still a limiting factor for UEFTC’s application on datasets of large sample sizes.

Prioritizing many tables from biological data exposed limitations of other methods that may not be obvious in simulation studies. For example, GKT performed well in our simulation studies. However, along with FOI and CE, GKT is highly sensitive to outliers and their top patterns are close to constant functions, uninteresting both statistically and mathematically.

We further discovered anti-co-methylation patterns from DNA methylation data. This is striking biologically, as it may require orchestrated regulation of both methylation and demethylation to take place at CpG sites in the vicinity of the same gene, in contrast to co-methylation where CpG sites are all methylated or all demethylated. As the observed anti-co-methylation depends on disease status, this contrast may offer a high quality signal to be used as a disease biomarker.

## 5 Conclusion

In summary, we have presented a statistical test UEFTC for discrete function inference without assuming a parametric mathematical model. As demonstrated by simulation studies and in biological pattern discovery, UEFTC exhibits the best combined performance in inferring function strength and direction, among other options to solve the same problem. Unique from other related tests using observed marginal distributions or counts for the null population, UEFTC instead imposes embedded uniform squares. This design has not only reduced function direction biases due to variable cardinality or table shape, but also enhanced robustness to outliers often seen in data with large dynamic ranges. We expect to see applications of exact model-free function tests on pilot studies which demand accurate *P*-value calculation, so as to generate hypotheses on causal relationships in an ocean of overwhelming possibilities, populated by the ever increasing throughput of modern data acquisition such as in biology.

## Supplementary Material

btaf121_Supplementary_Data

## Data Availability

The cause-effect pairs data were downloaded from https://webdav.tuebingen.mpg.de/cause-effect/ The yeast RNA-seq dataset was downloaded using NCBI GEO accession GSE158318. The EED methylation and RNA-seq data were downloaded using NCBI GEO accessions GSE157914 and GSE159495, respectively.
