# Supplementary material for: Exact model-free function inference using uniform marginal counts for null population
Source: Bioinformatics. 2025 Mar 20;41(4):btaf121. doi: 10.1093/bioinformatics/btaf121 (PMC11972114; doi:10.1093/bioinformatics/btaf121)
Supplement: btaf121_Supplementary_Data [file btaf121_supplementary_data.zip › supplement-2025-03-11.pdf]

Exact model-free function inference using uniform marginal counts for null population

Yiyi Li and Mingzhou Song

Supplementary Materials

A The proof of Theorem 1

Lemma 1. *Let $a_1 \le \cdots \le a_n$ be n sorted non-negative integers with sum $N = \sum_{i=1}^{n} a_i$. For the integer division N/n, let $D = \lfloor N/n \rfloor$ be the quotient and $p = N \bmod n$ be the remainder. We define n integers $D_1, \ldots, D_n$ to be*

$$\underbrace{D, \ldots, D}_{(n-p)\ \text{times}}, \underbrace{D+1, \ldots, D+1}_{p\ \text{times}} \tag{S1}$$

It must be true that $a_1 \le D_1$ and $a_n \ge D_n$.

Proof. (By contradiction)

Suppose $a_1 > D_1$. Since a_1 is an integer and the remainder p is at most $n-1$, we would have $a_1 \ge D_1 + 1 = D + 1$. As other a_i's are no less than a_1, it would imply

$$N = \sum_{i=1}^{n} a_i \ge n a_1 \ge n(D+1) \tag{S2}$$

As $p = N \bmod n$, we have $0 \le p < n$ and $n - p > 0$, suggesting

$$N = \sum_{i}^{n} D_i = (n-p)D + p(D+1) < n(D+1) \tag{S3}$$

The above two inequalities lead to a mathematical conflict between $N \ge n(D+1)$ and $N < n(D+1)$. Thus, instead of $a_1 > D_1$, we must have the opposite relationship $a_1 \le D_1$ true.

Suppose $a_n < D_n$. We show conflicts in two cases:

Case 1. $p = 0$**.** Since $D_n = D = N/n$, we would have $a_1 \le \cdots \le a_n < D$, implying

$$N = \sum_{i=1}^{n} a_i < nD = N \tag{S4}$$

leading to a mathematical conflict of N being less than N.

Case 2. $p > 0$**.** We would have $a_n \le D_n - 1 = D$. As all other a_i's are no larger than a_n, it would suggest

$$N = \sum_{i=1}^{n} a_i \le nD \tag{S5}$$

However, $p > 0$ implies

$$N = \sum_{i=1}^{n} D_i = (n-p)D + p(D+1) > nD \tag{S6}$$

leading to a mathematical conflict between $N \le nD$ and $N > nD$.

Therefore, inequality $a_n < D_n$ is incorrect and the opposite relationship $a_n \ge D_n$ must be true. □

Lemma 2. *Let $a_1 \le \cdots \le a_n$ be $n \ge 2$ sorted non-negative integers with sum $N = \sum_{i=1}^{n} a_i$. Let $D = \lfloor N/n \rfloor$ be the integer division quotient. Let $p = N \bmod n$ be the integer division remainder. We define n integers $D_1, \ldots, D_n$ by*

$$D_i = \begin{cases} D & i = 1, \ldots, n-p \\ D+1 & i = n-p+1, \ldots, n \end{cases} \tag{S7}$$

and n integers $b_1, \ldots, b_n$ by

$$b_i = a_i - D_i, \quad i = 1, \ldots, n \tag{S8}$$

Then, one can find $1 \le q < n$ such that $b_1, \ldots, b_n$ are partitioned into a lower section of q non-positive numbers $b_1, \ldots, b_q \le 0$ and an upper section of $n-q$ non-negative numbers $b_{q+1}, \ldots, b_n \ge 0$.

Proof. As a_i's are sorted, we have $b_1 \le \cdots \le b_{n-p}$ and $b_{n-p+1} \le \cdots \le b_n$. However, b_i's are not necessarily sorted because it is possible that $b_{n-p} > b_{n-p+1}$. Still, we argue that b_i's signs cannot switch from positive to negative as i increases. Such a transition would be possible only from a positive $b_{n-p} > 0$ to a negative $b_{n-p+1} < 0$, but not at other places. As b_{n-p} is an integer, it must be true that $b_{n-p} \ge 1$, leading to $a_{n-p} \ge D+1$. Since $a_{n-p+1} \ge a_{n-p}$ by a_i's definition, we have $a_{n-p+1} \ge D+1$, resulting in $b_{n-p+1} = a_{n-p+1} - (D+1) \ge 0$ not having a negative sign. Based on Lemma 1 and $n \ge 2$, $b_1 = a_1 - D_1 \le 0$ can always be in the lower section, and $b_n = a_n - D_n$ can always be put into the upper section. So neither section is empty. Therefore, we can always choose a q from 1 to $n-1$ to partition b_i's into a lower section of q non-positive numbers and an upper section of $n-q$ non-negative numbers. □

Lemma 3. *Let $a_1 \le \cdots \le a_n$ be n sorted non-negative integers with sum $N = \sum_{i=1}^{n} a_i$. Let $D = \lfloor N/n \rfloor$ be the integer division quotient. Let $p = N \bmod n$ be the integer division remainder. Then the sum of integer squares has the following lower bound:*

$$\sum_{i=1}^{n} a_i^2 \ge (n-p)D^2 + p(D+1)^2 \tag{S9}$$

The equality holds if and only if $a_1, \ldots, a_n$ are equal to $\underbrace{D, \ldots, D}_{(n-p) \text{ times}}, \underbrace{D+1, \ldots, D+1}_{p \text{ times}}$.

Proof. We define integers $D_1, \ldots, D_n$ by Eq. (S7) and integers $b_1, \ldots, b_n$ by Eq. (S8), which implies immediately

$$\sum_{i=1}^{n} b_i = \sum_{i=1}^{n} a_i - \sum_{i=1}^{n} D_i = N - N = 0 \tag{S10}$$

The sum of integer squares can be bounded as follows:

$$\sum_{i=1}^{n} a_i^2 = \sum_{i=1}^{n} (D_i + b_i)^2 \tag{S11}$$

$$= \sum_{i=1}^{n} D_i^2 + \sum_{i=1}^{n} b_i^2 + 2 \sum_{i=1}^{n} b_i D_i \tag{S12}$$

$$\geq \sum_{i=1}^{n} D_i^2 + 2 \sum_{i=1}^{n} b_i D_i \tag{S13}$$

By Lemma 2, we can find q such that $b_1, \ldots, b_q \leq 0$, $b_{q+1}, \ldots, b_n \geq 0$. By Lemma 1, we have $b_n = a_n - D_n \geq 0$, so that we can always choose $q < n$ to place b_n in the upper non-negative section. Therefore, we have two cases of either $1 \leq q \leq n - p$ or $n - p + 1 \leq q \leq n - 1$. If $p = 0$, only the first case applies. Next, we show in both cases that

$$\sum_{i=1}^{n} b_i D_i \geq 0 \tag{S14}$$

Case 1. $1 \leq q \leq n - p$**.** To cover the case of $p = 0$, we introduce $b_{n+1} = 0$ and $D_{n+1} = D + 1$.

$$\sum_{i=1}^{n} b_i D_i = \sum_{i=1}^{n+1} b_i D_i \tag{S15}$$

$$= \sum_{i=1}^{n-p} b_i D_i + \sum_{i=n-p+1}^{n+1} b_i D_i \tag{S16}$$

$$= D \sum_{i=1}^{n-p} b_i + (D + 1) \sum_{i=n-p+1}^{n+1} b_i \tag{S17}$$

$$= D \sum_{i=1}^{n+1} b_i + \sum_{i=n-p+1}^{n+1} b_i \tag{S18}$$

$$= \sum_{i=n-p+1}^{n+1} b_i \qquad (\because \text{Eq. } (S10);\ b_{n+1} = 0) \tag{S19}$$

$$\geq 0 \qquad (\because b_{n-p+1}, \ldots, b_n \geq 0;\ b_{n+1} = 0) \tag{S20}$$

Case 2. $n - p + 1 \le q \le n - 1$.

$$\sum_{i=1}^{n} b_i D_i = \sum_{i=1}^{n-p} b_i D_i + \sum_{i=n-p+1}^{q} b_i D_i + \sum_{i=q+1}^{n} b_i D_i \tag{S21}$$

$$= D \sum_{i=1}^{n-p} b_i + (D+1) \sum_{i=n-p+1}^{q} b_i + (D+1) \sum_{i=q+1}^{n} b_i \tag{S22}$$

$$= D \sum_{i=1}^{n} b_i + \sum_{i=n-p+1}^{q} b_i + \sum_{i=q+1}^{n} b_i \tag{S23}$$

$$= \sum_{i=n-p+1}^{q} b_i + \sum_{i=q+1}^{n} b_i \tag{S24}$$

$$= 0 - \sum_{i=1}^{n-p} b_i \qquad (\because \text{Eq. } (S10)) \tag{S25}$$

$$\ge 0 \qquad (\because b_1, \ldots, b_{n-p} \le 0) \tag{S26}$$

Thus, we have shown $\sum_{i=1}^{n} b_i D_i \ge 0$. Plugging the inequality into Eq. ()S13), we can establish the lower bound of integer sum of squares

$$\sum_{i=1}^{n} a_i^2 \ge \sum_{i=1}^{n} D_i^2 + 2 \sum_{i=1}^{n} b_i D_i \ge \sum_{i=1}^{n} D_i^2 = (n-p)D^2 + p(D+1)^2 \tag{S27}$$

thus proving Inequality (S9) stated in the lemma.

In both cases above, it is evident that the lower bound of 0 for $\sum_{i=1}^{n} b_i D_i$ can be achieved if and only if $b_i = 0$ for all $i = 1, \ldots, n$. That implies $a_i = D_i$ for $i = 1, \ldots, n$ being both sufficient and necessary for

$$\sum_{i=1}^{n} a_i^2 = \sum_{i=1}^{n} D_i^2 = (n-p)D^2 + p(D+1)^2 \tag{S28}$$

which proves the second statement in the lemma. □

Lemma 4. *Let A be an $r \times s$ contingency table of sample size N. Given row sums $n_{i\cdot}$ ($i = 1, \ldots, r$) of landscape tables, column sums $n_{\cdot j}$ ($j = 1, \ldots, s$) of portrait tables, the upper bounds of the FunChisq test statistic for A are*

$$\max \frac{\chi_f^2(A)}{s} = \begin{cases} N - \sum_{i=1}^{r} \dfrac{n_{i\cdot}^2}{N} & r < s \\ N - \sum_{j=1}^{s} \dfrac{n_{\cdot j}^2}{N} & r \ge s \end{cases} \tag{S29}$$

Conditioned on the given marginal sums, the bounds are reached if and only if the column variable is a non-constant function of the row variable.

This lemma has been proved in Dr. Hien Nguyen's dissertation (Nguyen, 2018). The bounds, conditioned on given row or column sums, are not global maxima. Next, we examine the global upper bounds.

Theorem 1. *Let $\mathcal{A}$ be the set of all $r \times s$ tables of sample size N. Let $\mathcal{B}$ be a subset of $\mathcal{A}$ so that $\mathcal{B}$ contains those tables in $\mathcal{A}$ with uniform row and column sums. Both sets achieve the same maximum test statistic:*

$$\max_{B \in \mathcal{B}} \chi_f^2(B) = \max_{A \in \mathcal{A}} \chi_f^2(A) \tag{S30}$$

Proof. We prove the theorem in two cases: one for landscape tables and one for square and portrait tables.

Case 1. Landscape tables ($r < s$). By definition of $\mathcal{B}$ where row sums of any table are R^0, we can always find a function table $B^* \in \mathcal{B}$

$$B^* = \begin{bmatrix} R_1 & 0 & \cdots & 0 & 0 & \cdots & 0 \\ 0 & R_2 & \cdots & 0 & 0 & \cdots & 0 \\ 0 & 0 & \ddots & 0 & 0 & \cdots & 0 \\ 0 & 0 & \cdots & R_r & 0 & \cdots & 0 \end{bmatrix} \tag{S31}$$

where

$$B^*[i,j] = \begin{cases} R_i & 1 \le i = j \le r \\ 0 & \text{otherwise} \end{cases} \tag{S32}$$

to attain the maximum statistic on $\mathcal{B}$ by Lemma 4 for landscape tables

$$\max_{B \in \mathcal{B}} \frac{\chi_f^2(B)}{s} = \frac{\chi_f^2(B^*)}{s} = N - \sum_{i=1}^{r} \frac{R_i^2}{N} \tag{S33}$$

Also based on Lemma 4, we can obtain

$$\max_{A \in \mathcal{A}} \frac{\chi_f^2(A)}{s} = N - \min \sum_{i=1}^{r} \frac{n_{i\cdot}^2}{N} \tag{S34}$$

By Lemma 3, the uniform row sums in $R^0 = (R_1, \ldots, R_r)$ as defined by Eq. (5) minimize the sum of squares:

$$\min \sum_{i=1}^{r} n_{i\cdot}^2 = \sum_{i=1}^{r} R_i^2 \tag{S35}$$

implying

$$\max_{A \in \mathcal{A}} \frac{\chi_f^2(A)}{s} = N - \min \sum_{i=1}^{r} \frac{n_{i\cdot}^2}{N} = N - \sum_{i=1}^{r} \frac{R_i^2}{N} \tag{S36}$$

Combining Eqs. (S33) and (S36), we arrive at

$$\max_{B \in \mathcal{B}} \chi_f^2(B) = \max_{A \in \mathcal{A}} \chi_f^2(A) \tag{S37}$$

proving the theorem for landscape tables.

Case 2. Portrait or square tables ($r \geq s$). Using similar arguments above, we can prove the theorem for non-landscape tables.

By definition of $\mathcal{B}$ where column sums of any table are C^0, we can always find a function table $B^* \in \mathcal{B}$

$$B^* = \begin{bmatrix} C_1 & 0 & \cdots & 0 \\ 0 & C_2 & \cdots & 0 \\ 0 & 0 & \ddots & 0 \\ 0 & 0 & \cdots & C_s \\ 0 & 0 & 0 & 0 \\ \vdots & \vdots & \vdots & \vdots \\ 0 & 0 & 0 & 0 \end{bmatrix} \tag{S38}$$

where

$$B^*[i,j] = \begin{cases} C_j & 1 \leq i = j \leq s \\ 0 & \text{otherwise} \end{cases} \tag{S39}$$

to attain the maximum statistic on $\mathcal{B}$ by Lemma 4 for square or portrait tables

$$\max_{B\in\mathcal{B}} \frac{\chi_f^2(B)}{s} = \frac{\chi_f^2(B^*)}{s} = N - \sum_{j=1}^{s} \frac{C_j^2}{N} \tag{S40}$$

Also based on Lemma 4, we can derive

$$\max_{A\in\mathcal{A}} \frac{\chi_f^2(A)}{s} = N - \min \sum_{j=1}^{s} \frac{n_{\cdot j}^2}{N} \tag{S41}$$

By Lemma 3, the uniform column sums in $C^0 = (C_1, \ldots, C_s)$ as defined by Eq. (6) minimize the sum of squares:

$$\min \sum_{j=1}^{s} n_{\cdot j}^2 = \sum_{j=1}^{s} C_j^2 \tag{S42}$$

suggesting

$$\max_{A\in\mathcal{A}} \frac{\chi_f^2(A)}{s} = N - \min \sum_{j=1}^{s} \frac{n_{\cdot j}^2}{N} = N - \sum_{j=1}^{s} \frac{C_j^2}{N} \tag{S43}$$

Integrating Eqs. (S40) and (S43), we obtain

$$\max_{B\in\mathcal{B}} \chi_f^2(B) = \max_{A\in\mathcal{A}} \chi_f^2(A) \tag{S44}$$

proving the theorem for square and portrait tables.

Therefore, the maximum of FunChisq test statistic $\chi_f^2(A)$ among tables in $\mathcal{A}$ with given sample and table sizes can always be reached by some table in the uniform null population $\mathcal{B}$ that we have designed. □

B Designed algorithms for fast p-value calculation

To cover null population $\mathcal{B}$, we build a directed acyclic graph T with layers $l = 0, \ldots, r$ (Figure S1). A node in layer l represents a subset of $l \times s$ sub-tables, sharing and labeled by common column sums $(S_{l,1}, \ldots, S_{l,s})$ unique within layer l. Each sub-table is the last l rows in some null table $B \in \mathcal{B}$. Layer $l = r$ contains only one node v_r, called *source* with label C^0, covering all tables in $\mathcal{B}$. Layer $l = 0$ has only one node v_0, called *sink*, labeled by an s-dimension zero vector $(0, \ldots, 0)$, representing a dummy sub-table of $l = 0$ row. A directed edge (v, w) must go from a parent node v in layer l to a valid child node w in layer $l-1$, where $S_{l-1,j} \leq S_{l,j}$ for $j = 1, \ldots, s$. $S_{l,j} - S_{l-1,j}$ enumerates entry $B[l, j]$ in a null table B. Taking all s such values ($j = 1, \ldots, s$) together, an edge from layer l to $l-1$ enumerates row l for B. w in layer $l-1$ can be a common child of multiple parents in layer l, so T is not a tree.

$$
\begin{array}{ll}
\text{Layer } r\text{:} & \text{source } v_r\text{: } C^0 = (C_1, \ldots, C_s) \\
 & \swarrow \downarrow \searrow \\
 & \vdots \\
\text{Layer } l\text{:} & \cdots, \text{parent } v\text{: } (S_{l,1}, \ldots, S_{l,s}), \cdots \\
 & \downarrow \\
\text{Layer } l-1\text{:} & \cdots, \text{child } w\text{: } (S_{l-1,1}, \ldots, S_{l-1,s}), \cdots \\
 & \vdots \\
 & \searrow \downarrow \swarrow \\
\text{Layer } 0\text{:} & \text{sink } v_0\text{: } (0, \ldots, 0)
\end{array}
$$

Supplementary Figure S1: The graph covering all tables in null population $\mathcal{B}$.

Given a parent v in layer l and a child w in layer $l-1$, and w's column sums $(S_{l-1,1}, \ldots, S_{l-1,j-1})$ preceding column j, $S_{l-1,j}$ (sum of column j in w) is constrained by

$$
\max\left\{0,\ S_{l,j} - R_l + \sum_{i=1}^{j-1}(S_{l,i} - S_{l-1,i})\right\} \leq S_{l-1,j} \leq \min\left\{S_{l,j},\ \sum_{i=1}^{l-1} R_i - \sum_{i=1}^{j-1} S_{l-1,i}\right\} \tag{S45}
$$

useful in enumerating valid children in layer $l-1$ from a parent in layer l.

Given node v_l in layer l and node v_{l-1} in layer $l-1$, we define the weight of edge (v_l, v_{l-1}) by

$$
\text{edgeWeight}(v_l, v_{l-1}) = \sum_{j=1}^{s} \frac{(S_{l,j} - S_{l-1,j})^2}{R_l} \tag{S46}
$$

which is the edge's contribution to test statistic χ_f^2. We also define the length of edge (v_l, v_{l-1}) by

$$
\text{edgeLength}(v_l, v_{l-1}) = \frac{R_l!}{\prod_{j=1}^{s}(S_{l,j} - S_{l-1,j})!} \tag{S47}
$$

contributing to the hypergeometric probability of a null table.

By the definition of edges, a path $\pi(v_l)$ from source v_r to a node v_l in layer l represents a *specific* sub-table with $r - l$ rows. We define the weight of path $\pi(v_l)$ to be the test statistic of the sub-table:

$$\text{pathWeight}(\pi(v_l)) = \sum_{i=l+1}^{r} \frac{(S_{i,1} - S_{i-1,1})^2 + \cdots + (S_{i,s} - S_{i-1,s})^2}{R_i} \tag{S48}$$

We define the length of path $\pi(v_l)$ to be the null probability of the sub-table:

$$\text{pathLength}(\pi(v_l)) = \prod_{i=l+1}^{r} \frac{R_i!}{\prod_{j=1}^{s}(S_{i,j} - S_{i-1,j})!} \tag{S49}$$

All paths from v to sink v_0 represent all possible sub-tables spanning row 1 to l, constrained by row sums $R_1, \ldots, R_l$ and column sums $S_{l,1}, \ldots, S_{l,s}$. We call the sum of their null probabilities

$$\text{sumPathLength}(v, v_0) = \frac{\left(\sum_{i=1}^{l} R_i\right)!}{\prod_{j=1}^{s}(S_{l,j}!)} \tag{S50}$$

which efficiently accumulates the p-value, if each of these sub-tables will lead to a full table (with row $l + 1$ to r fixed) that must be included in p-value calculation.

Algorithm S1 buildNullGraph constructs a directed graph of a subspace of tables in the null population $\mathcal{B}$. Sub-tables which are stronger than the observed tables are trimmed to save space, but they are all accounted for in the p-value calculated. It grows the graph from source v_r to sink v_0. The built graph contains tight bounds of the test statistic associated with each sub-table via dynamic programming to avoid exhaustive table enumeration in the next stage.

Algorithm S2 computeExactPvalue returns the exact p-value of an observed test statistic χ_f^2. It traverses the null table space built by Alg. S1 buildNullGraph to sum up exact probabilities of null tables (paths from source to sink) of no less than observed test statistic. It also returns mathematical bounds for the observed statistic. These tight bounds are achievable by specific tables in the null population $\mathcal{B}$ and will be used for continuity correction.

Algorithm S3 UEFT(O) computes the exact p-value of an observed table O by calling Alg. S2 for the fast and exact p-value calculation.

Algorithm S4 UEFTC(O) performs p-value continuity correction based on three p-values computed on null tables with test statistics surrounding the observed test statistic $\chi_f^2(O)$.

Algorithm S1 buildNullGraph(χ^2_f, R^0, C^0, Equal)

Input:

χ^2_f: observed test statistic of $r \times s$ table O; R^0, C^0: null row and column sum vectors
Equal: true for $P = \sum_{B:\chi^2_f(B) \geq \chi^2_f(O)} \Pr(B)$, false for $P = \sum_{B:\chi^2_f(B) > \chi^2_f(O)} \Pr(B)$

Output: T: a directed graph with upper and lower bounds for each node

```
// Step 1: Build a graph to represent the null table population
Initialize source node v_r to be labeled C^0
for layer l ← r downto 1 do                                  ▹ Grow the graph from source v_r to sink v_0
    for each node v in layer l do
        minStat(v) ← min(pathWeight(π(v)))
        if minStat(v) < χ_f^2 and Equal = true or minStat(v) ≤ χ_f^2 and Equal = false then
            Enumerate child nodes w's in layer l − 1 constrained by Eq. (S45)
            for each child node w of v do
                Update test statistic lower bound: minStat(w) ← min(pathWeight(π(w)))
// Step 2: Obtain tight bounds by dynamic programming.
LB(v_0) = UB(v_0) = 0                                        ▹ Set lower and upper bounds for sink v_0
for layer l ← 1 upto r do
    for each node v in layer l do
        LB(v) = min_{u∈v's parents in layer l+1} LB(u) + edgeWeight(u, v)
        UB(v) = max_{u∈v's parents in layer l+1} UB(u) + edgeWeight(u, v)
return T
```

Algorithm S2 computeExactPvalue(χ_f^2, R^0, C^0, Null graph T, Equal)

Input:

χ_f^2: observed test statistic; R^0, C^0: null row and column sum vectors
T: graph representing the null population
Equal: true for $P = \sum_{B:\chi_f^2(B) \geq \chi_f^2(O)} \Pr(B)$, false for $P = \sum_{B:\chi_f^2(B) > \chi_f^2(O)} \Pr(B)$

Output: P: exact p-value; LS: left statistic; RS: right statistic

$P \leftarrow 0$, $LS \leftarrow 0$, $RS \leftarrow \infty$
▹ **Traverse graph T to find tight statistic bounds and p-value**
for layer $l \leftarrow r$ downto 1 in graph T **do**
  for each node v in layer l **do**
    for each path $\pi(u)$ reaching node v via edge (u, v) **do**
      $UB(\pi(v)) \leftarrow$ pathWeight($\pi(u)$) + $UB(v)$ ▹ Get upper bound of path π_l
      $LB(\pi(v)) \leftarrow$ pathWeight($\pi(u)$) + $LB(v)$ ▹ Get lower bound of path π_l
      if $UB(\pi(v)) \leq \chi_f^2$ **and** $UB(\pi(v)) > LS$ **then**
        $LS = UB(\pi(v))$ ▹ Update left statistic
      if $LB(\pi(v)) \geq \chi_f^2$ **and** $LB(\pi(v)) < RS$ **then**
        $RS = LB(\pi(v))$ ▹ Update right statistic
      if ($UB(\pi(v)) < \chi_f^2$ **and** Equal = true) **or** ($UB(\pi(v)) \leq \chi_f^2$ **and** Equal = false) **then**
        continue ▹ Abandon all branches in layers below
      else if $LB(\pi(v)) \geq \chi_f^2$ **and** Equal = true **or** $LB(\pi(v)) > \chi_f^2$ **and** Equal = false **then**
        $P = P$ + pathLength($\pi(v)$) $\cdot$ sumPathLength(v, v_0) ▹ Accumulate p-value
      else ▹ Extend the path to child nodes
        for each child node w of v **do**
          Append node w to path $\pi(v)$ to create path $\pi(w)$
          pathWeight($\pi(w)$) = pathWeight($\pi(v)$) + edgeWeight(v, w)
          pathLength($\pi(w)$) = pathLength($\pi(v)$) $\cdot$ edgeLength(v, w)
return P, LS, RS

Algorithm S3 Uniform Exact Function Test UEFT(O)

Input: O: observed $r \times s$ contingency table
Output: P: exact p-value of table O by UEFT

1: Uniform null row sums $R^0 \leftarrow (R_1, \ldots, R_r)$ by Eq. (5)
2: Uniform null column sums $C^0 \leftarrow (C_1, \ldots, C_s)$ by Eq. (6)
3: T = buildNullGraph($\chi_f^2(O), R^0, C^0$, true) ▹ Build a graph for the null table population
4: $\{P, -, -\}$ = computeExactPvalue(V, R^0, C^0, T, true) ▹ '−' ≡ ignored
5: **return** P

Algorithm S4 Uniform Exact Function Test with Continuity Correction UEFTC(O)

Input: O: observed $r \times s$ contingency table
Output: P_c: p-value of table O by UEFTC

1: Uniform null row sums $R^0 \leftarrow (R_1, \ldots, R_r)$ by Eq. (5)
2: Uniform null column sums $C^0 \leftarrow (C_1, \ldots, C_s)$ by Eq. (6)
3: T = buildNullGraph($\chi_f^2(O), R^0, C^0$, true) ▹ Build a graph for the null table population
4: $\{P_U(O), \chi_f^2(B_l), \chi_f^2(B_v)\}$ = computeExactPvalue($\chi_f^2(O), R^0, C^0, T$, true)
5: $\{P_U(B_l), -, -\}$ = computeExactPvalue($\chi_f^2(B_l), R^0, C^0, T$, true) ▹ '−' ≡ ignored
6: $\{P_U(B_w), -, -\}$ = computeExactPvalue($\chi_f^2(B_v), R^0, C^0, T$, false)
7: $P_c \leftarrow$ Perform continuity correction on p-value by Eq. (16)
8: **return** P_c as the continuity corrected p-value

B.1 Running time comparison

Although the worst-case time complexity for both UEFT and UEFTC tests are $O((rs)^N)$, they can be greatly sped up by the fast branch-and-bound algorithm (Alg. S2). We empirically evaluate the time efficiency of our fast algorithms. We simulated 100 tables of dimension 4×4, 5×3 and 3×5 with sample sizes ranging from 7 to 45. Then we applied the four exact methods including Fisher's exact test, UEFTC, UEFT and EFT to these tables and record the average running time of each table setup as shown in Supplementary Figure S2.

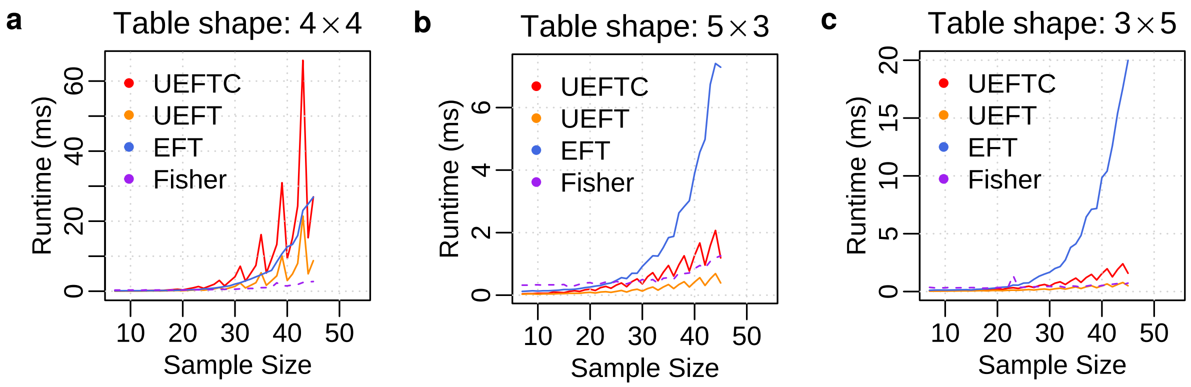

Supplementary Figure S2: Empirical time efficiency of three exact function tests. The horizontal axis is the sample size. The vertical axis is the average running time (ms) on 100 randomly generated (a) square, (b) portrait, and (c) landscape tables.

Although Fisher is fastest on square tables, UEFT and UEFTC are comparable to Fisher on non-squared tables and are more efficient then EFT. A main reason is that for non-square tables, the null population of UEFT and UEFTC are consisted of tables formed by smaller square tables and zero filled portions, rather than enumerating the full table size as done in EFT. Such change in the null population designed to improve statistical power also benefited the time efficiency.

As UEFTC calls Alg. S2 computeExactPvalue three times and UEFT invokes it only once, UEFTC is approximately three times slower than UEFT. As the sample size increases, the runtime of UEFTC and UEFT grows but with substantial fluctuations, likely due to whether the sample size N is a multiple of $\min(r, s)$.

C Function strength under various marginal distributions

Supplementary Table S1 is configurations used to generate results for Figure 5.

Supplementary Table S1: Simulation configurations for independent and functional tables.

	**Independent** $X \perp Y$	**Functional** $Y = f(X)$
Table size ($r \times s$)	3×3, 4×3, 3×4, 7×2, 2×7	
Sample size N	8, 12, 16, 20, 24, 28, 32, 36, 40, 44	
Noise level	No noise added	0.01, 0.15, 0.3, 0.45, 0.6, 0.75, 0.9
X (row) marginal distribution	Uniform, non-uniform	Uniform
Y (column) marginal distribution	Uniform, non-uniform	Not configurable
Number of tables per setup	1400	400
Total number of tables	280,000	140,000

We group the simulated independent tables by four marginal distribution types to evaluate the methods. The simulation configurations are described in Supplementary Tables S2, S3, S4, and S5.

When both row and column are uniformly distributed, all methods performed comparably (Supplementary Figure S3d). When the column is non-uniformly distributed, UEFTC obtained the second highest AUPR values in two cases, second highest AUROC in one case, and third highest AUROC once. UEFTC has reduced accuracy when row is non-uniform and column is uniform with the fourth highest AUROC and AUPR values.

Supplementary Table S2: Simulation configurations for independent and functional tables. Both row and column marginals are non-uniformly distributed.

	**Independent** $X \perp Y$	**Functional** $Y = f(X)$
Table size ($r \times s$)	3×3, 4×3, 3×4, 7×2, 2×7	
Sample size N	8, 12, 16, 20, 24, 28, 32, 36, 40, 44	
Noise level	No noise added	0.01, 0.15, 0.3, 0.45, 0.6, 0.75, 0.9
X (row) marginal distribution	non-uniform	Uniform
Y (column) marginal distribution	non-uniform	Not configurable
Number of tables per setup	2800	200
Total number of tables	140,000	70,000

Supplementary Table S3: Simulation configurations for independent and functional tables. Row marginal is non-uniformly and column marginal is uniformly distributed.

	**Independent** $X \perp Y$	**Functional** $Y = f(X)$
Table size ($r \times s$)	3×3, 4×3, 3×4, 7×2, 2×7	
Sample size N	8, 12, 16, 20, 24, 28, 32, 36, 40, 44	
Noise level	No noise added	0.01, 0.15, 0.3, 0.45, 0.6, 0.75, 0.9
X (row) marginal distribution	Non-uniform	Uniform
Y (column) marginal distribution	Uniform	Not configurable
Number of tables per setup	2800	200
Total number of tables	140,000	70,000

Supplementary Table S4: Simulation configurations for independent and functional tables. Row marginal is uniformly and column marginal is non-uniformly distributed.

	**Independent** $X \perp Y$	**Functional** $Y = f(X)$
Table size ($r \times s$)	3×3, 4×3, 3×4, 7×2, 2×7	
Sample size N	8, 12, 16, 20, 24, 28, 32, 36, 40, 44	
Noise level	No noise added	0.01, 0.15, 0.3, 0.45, 0.6, 0.75, 0.9
X (row) marginal distribution	Uniform	Uniform
Y (column) marginal distribution	Non-uniform	Not configurable
Number of tables per setup	2800	200
Total number of tables	140,000	70,000

Supplementary Table S5: Simulation configurations for independent and functional tables. Row and column marginals are uniformly distributed.

	**Independent** $X \perp Y$	**Functional** $Y = f(X)$
Table size ($r \times s$)	3×3, 4×3, 3×4, 7×2, 2×7	
Sample size N	8, 12, 16, 20, 24, 28, 32, 36, 40, 44	
Noise level	No noise added	0.01, 0.15, 0.3, 0.45, 0.6, 0.75, 0.9
X (row) marginal distribution	Uniform	Uniform
Y (column) marginal distribution	Uniform	Not configurable
Number of tables per setup	2800	200
Total number of tables	140,000	70,000

a. Non-uniform row and column marginals

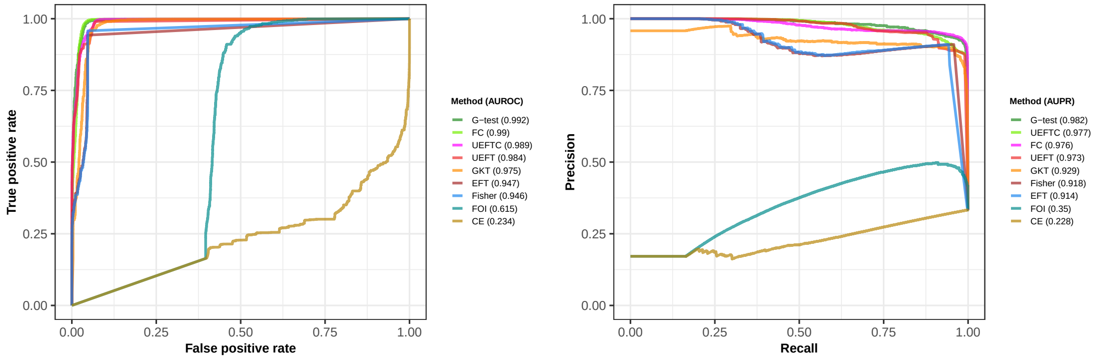

b. Non-uniform row and uniform column marginals

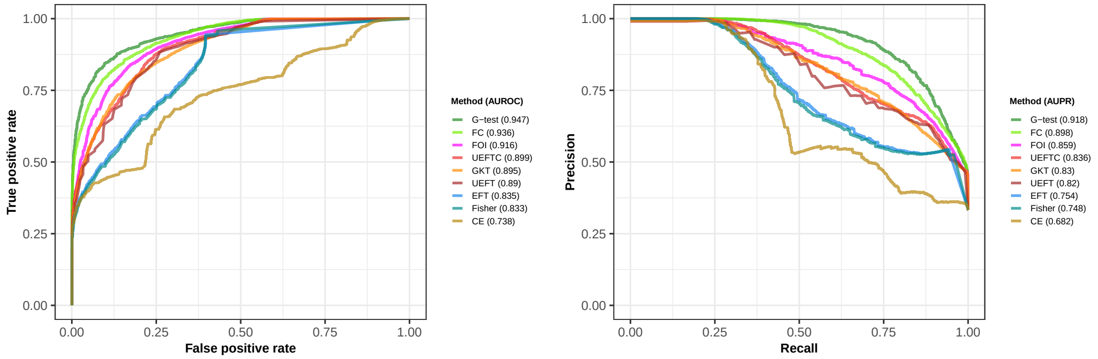

c. Uniform row and non-uniform column marginals

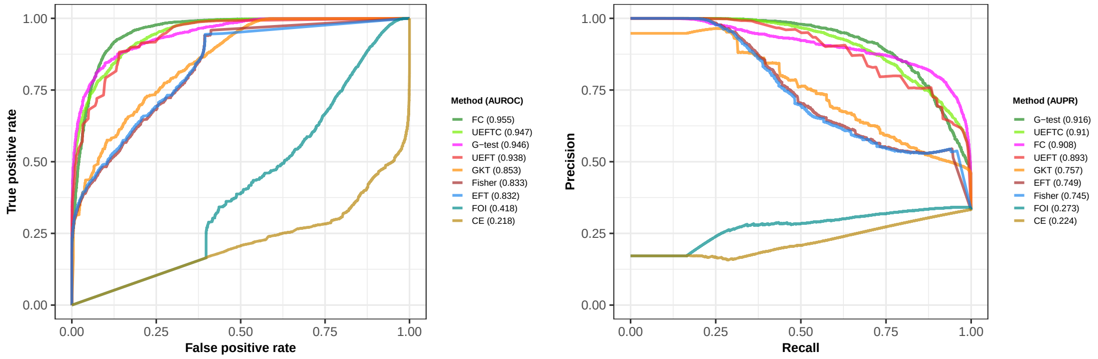

d. Uniform row and column marginals

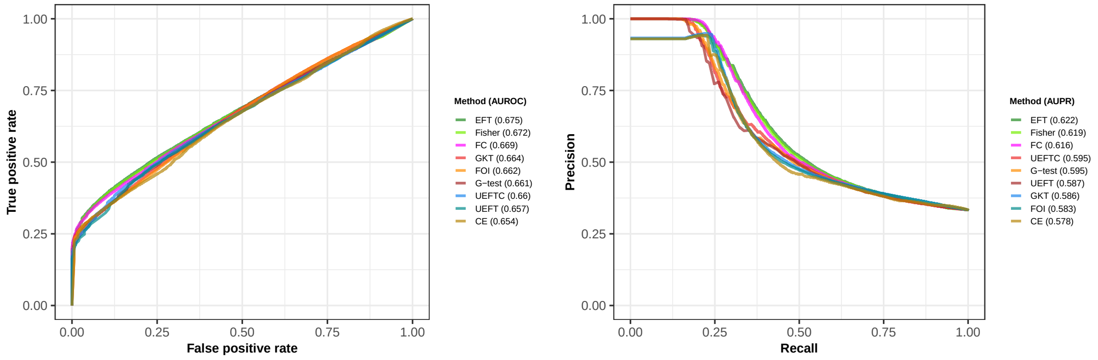

Supplementary Figure S3: Performance by marginal distributions of independent samples of nine methods on distinguishing functional from independent patterns. ROC and PR curves are shown for (a) non-uniform row and column, (b) non-uniform row and uniform column, (c) uniform row and non-uniform column, and (d) uniform row and column marginal distributions.

D Configurations for simulating tables to study statistical power and Type 1 error rate

Supplementary Tables S6 and S7 here are configurations used to generate results for Figure 6.

Supplementary Table S6: Simulation configurations for type 1 error and statistical power over sample sizes.

	**Independent** $X \perp Y$	**Functional** $Y = f(X)$
Table size ($r \times s$)	3×3, 4×3, 3×4, 7×2, 2×7	
Sample size N	8, 12, 16, 20, 24, 28, 32, 36, 40, 44	
Noise level	No noise added	0.01
X (row) marginal distribution	Uniform, non-uniform	Uniform
Y (column) marginal distribution	Uniform, non-uniform	Not configurable
Number of tables per setup	200	400
Total number of tables	40,000	20,000

Supplementary Table S7: Simulation configurations for statistical power over noise levels.

	**Independent** $X \perp Y$	**Functional** $Y = f(X)$
Table size ($r \times s$)	3×3, 4×3, 3×4, 7×2, 2×7	
Sample size N	40	
Noise level	No noise added	0.01, 0.15, 0.3, 0.45, 0.6, 0.75, 0.9
X (row) marginal distribution	Uniform, non-uniform	Uniform
Y (column) marginal distribution	Uniform, non-uniform	Not configurable
Number of tables per setup	1400	400
Total number of tables	28,000	14,000

References

Nguyen, H. H. (2018). *Inference of Functional Dependency via Asymmetric, Optimal, and Model-free Statistics*. PhD thesis, Department of Computer Science, New Mexico State University, Las Cruces, NM, USA.
